# Screening Cellular Feature Measurements for Image-Based Assay Development

**DOI:** 10.1177/1087057110370895

**Published:** 2010-08

**Authors:** David J. Logan, Anne E. Carpenter

**Affiliations:** Imaging Platform, The Broad Institute of MIT and Harvard, Cambridge, MA.

**Keywords:** high-throughput screening, high-content screening, image-based screening, open-source software, assay development

## Abstract

The typical “design” approach to image-based assay development involves choosing measurements that are likely to correlate with the phenotype of interest, based on the researcher’s intuition and knowledge of image analysis. An alternate “screening” approach is to measure a large number of cellular features and systematically test each feature to identify those that are best able to distinguish positive and negative controls while taking precautions to avoid overfitting the available data. The cell measurement software the authors previously developed, CellProfiler, makes both approaches straightforward, easing the process of assay development. Here, they demonstrate the use of the screening approach to image assay development to select the best measures for scoring publicly available image sets of 2 cytoplasm-to-nucleus translocation assays and 2 Transfluor assays. The authors present the resulting assay quality measures as a baseline for future algorithm comparisons, and all software, methods, and images they present are freely available.

## Introduction

Image-based screening is becoming increasingly widespread and productive in both commercial and academic research environments, at both primary and secondary screening phases. It is being used for a wide variety of scientific objectives such as drug discovery, chemical probe discovery, and functional genomics using RNA interference-treated samples.^1-4^ Biologists have long depended on microscopy-based assays to study the mechanisms of biological processes, and the demand to move new image-based assays from low throughput to high throughput is growing substantially.

Most image-based high-throughput screening assays are currently analyzed using commercial instrumentation with bundled software, usually designed to score assays of particular pharmaceutical interest. In general, these assays are designed to produce a single numerical output per well. This approach simplifies the process of screening for the end user and streamlines high-content screening (HCS) so that it can approach the speed of traditional high-throughput assays. This strategy of simplification allows high-throughput screening (HTS) experts familiar with other screening modalities to transfer their knowledge to HCS.

Developing approaches to quantify novel assays using image analysis often remains a time-consuming challenge. Although developing a routine for a new assay is not always straightforward, several options exist: (1) write and test raw image analysis code from scratch, but this is time-consuming and requires a programmer with expertise in image analysis; (2) adapt existing assay-specific software; (3) force existing software to output additional measures of interest and explore those measures, but this sometimes requires processing images through several different assay-specific routines and combining the measurements; and (4) test multiple software packages, but this is rarely feasible due to the costs and the amount of time required to optimize each software package for the assay. In addition, proprietary software usually conceals the underlying algorithms so that when differences occur between software packages, it is unclear whether the algorithms themselves or slight differences in settings yielded the observed differences. Thus, the current approach to image-based assay development requires significant time, expertise, and/or expense to adapt to a novel assay.

An alternative to these approaches is to use machine learning to score images of individual cells or cell populations based on a combination of multiple features extracted from the cells. Although machine-learning approaches are not yet widespread for HCS, they have been shown useful for discerning complex phenotypes in many cases,^5-11^ and we are successfully using it for roughly half of the screens in our laboratory.^12^ There are some situations, however, in which a machine-learning approach is not suited for an assay. First, machine-learning algorithms must often be trained separately for each experimental batch to perform well; in some unfortunate cases, variations may be significant enough as to require separate training for each plate. This can be quite time-consuming as compared to normalizing a single feature across batches or setting thresholds for a single feature for each batch. Aside from this practical concern, a machine-learning approach may be scientifically undesirable: positive controls used to train the algorithm may exhibit more than just the perturbation of interest; consequently, the cells may also display other morphological changes that are not relevant to the biological process being studied. In contrast, scoring the assay using a single feature of known biological relevance affords the researcher more control over the phenotype to be scored. Thus, even though machine learning may score a phenotype more accurately in many cases, there are situations in which scoring the assay with a single, well-understood feature is preferable.

Here, we describe a screening approach that can improve image assay development when scoring assays based on a single feature.

## Materials and Methods

Images used in this work are publicly available and were graciously compiled by Ilya Ravkin (http://www.ravkin.net/SBS/Image-Library.htm). There are 4 image sets, 2 for the Transfluor assay (called CompuCyte and Roche) and 2 for the cytoplasm-to-nucleus assay (called BioImage and Vitra). These images are also freely available at the Broad Bioimage Benchmark Collection (http://www.broadinstitute.org/bbbc).

**Fig. 1.**
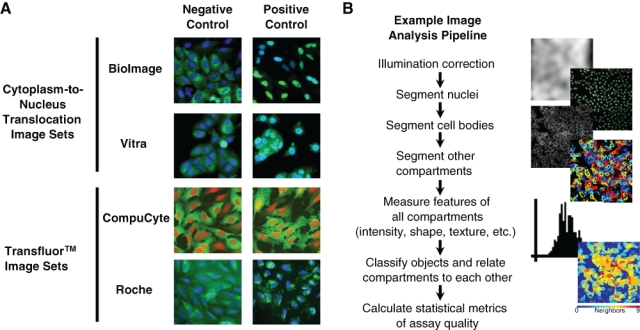
Image assays and image analysis pipeline design. (**A**) Two representative images from each of the 4 image sets are shown: 1 positive and 1 negative control. Each has a red or blue channel (DNA stain) and a green channel (labeling the molecule of interest). (**B**) A schematic pipeline, displaying typical steps of the image analysis pipeline.

The open-source cell image analysis software used here, CellProfiler, is freely available at http://www.cellprofiler.org.^13^ An image analysis pipeline (a serial set of image analysis algorithms) was constructed for each assay in version 2.0 of CellProfiler. This standard pipeline (**Fig. 1B**) was adjusted appropriately for each image set to load the images and metadata (e.g., plate-well identification and sample metadata, including compound, dose and positive/negative control status, and cell type), segment regions of interest, measure features, and export the resulting data. To correct for persistent illumination variations across all images (due to many possible sources, including optical hardware irregularities, illumination patterns, or shading), we performed illumination correction with a separate short CellProfiler pipeline. In brief, all images for each individual channel are summed, and then the resultant image is smoothed with a large median filter (~150 × 150 pixels) to construct illumination functions. This illumination function is then used by the main analysis pipeline: each raw image is divided by the illumination function before subsequent processing. Tutorials for creating and modifying CellProfiler pipelines are available online and in print,^14,15^ and the pipelines used in this study are freely available at the Broad Bioimage Benchmark Collection (http://www.broadinstitute.org/bbbc).

Image processing was conducted on a single node of the Broad computing cluster with these specifications: 64-bit AMD processor, SUSE Linux V10.1, Quad-Core AMD Opteron Processor 8356 at 2.3 GHz, 512 KB L2 cache, and 16 GB RAM. Note, however, that processing times on an Apple MacBook Pro with a 2.5-GHz processor and 4-GB 667-MHz RAM were typically within a factor of 1.5 of the cluster machine.

## Results

In our own laboratory, when a machine learning approach is not appropriate, we have typically scored image-based cellular assays using the design approach, using image analysis expertise to select or create a feature to be measured by our open-source high-throughput cell image analysis software, CellProfiler.^16-26^ In our experience, designing measurements to suit a particular new assay is fruitful. Still, we hypothesized that, like screening versus design approaches to identify drug leads, a screening approach to choose image-based assay features might be less time, resource, and expertise consuming in at least some situations. We set out to develop and test this approach.

Experienced screeners are well aware that the success of any screening approach is determined both by the design of the assay and by the quality of the library being screened. Correspondingly, screening to find an optimal measured feature to use for scoring a particular high-content assay is highly dependent on having a good library of features. This library must contain features that adequately represent the cellular morphology of interest in the assay. Flexible, modular software is needed for this purpose; CellProfiler meets these criteria by offering the use of numerous algorithms and optional settings, and it measures a large number of features, including the size and shape of each cell and subcellular compartment, as well as the intensity and texture of each stain within each cell and subcellular compartment.^27^ For a typical assay, this can yield roughly 500 to 1000 measured features per cell.

Although we call this method a “screening” approach, in reality, we do not screen an identical library of assay features for every new assay. Rather, we typically add several potentially useful, designed features to the library for each given assay. This strategy is analogous, in the screening world, to adding a sublibrary of chemical compounds thought to be more specific to the target class of interest, in addition to screening a large, diverse chemical library. The following exemplifies this principle. The standard image analysis pipeline records the mean and median of each measured cellular feature for all cells in an image. However, for some assays, the mean or median of per cell measurements is not the most useful metric. Rather, other features derived from the raw per cell measurements (called “derived features”) can be quite powerful. Derived features take advantage of individual cell data and include calculations such as ratios of features or the percentage of cells above a threshold. The framework of CellProfiler allows the researcher to use his or her intuition and knowledge to easily derive features and add them to the feature library, without computer programming. This process involves, for example, choosing which features to ratio or which threshold to apply to a feature. Currently, derived features can be added one by one to the feature library to be screened in CellProfiler using modules such as *Calculate Math* and *Classify Objects*. It is feasible to extend CellProfiler to record hundreds of thousands of features, for example, to count the percentage of cells in each sample above a particular threshold for *every* measured feature or to calculate a huge variety of ratios of features. This would add to the feature library but also would affect the rate of false-positive features scored as relevant to the assay, in addition to requiring more intense computing resources. Human intuition is therefore still highly valuable in choosing what features to include in the library so as to minimize the chances of overfitting the available data and/or generating false positives, as we discuss later.

For this experiment, we configured CellProfiler to identify and measure cells and subcellular compartments and added the *Calculate Statistics* module to the image-processing pipeline to calculate various measures of assay performance, which will be used as the basis for selecting a feature for the assay. These assay performance measures currently include the Z′ factor and the V factor. The Z′ factor indicates how well separated the positive and negative controls are, given the variation present in both populations.^28^ The V factor, by contrast, capitalizes on all the data along a dose-response curve rather than just the positive and negative controls alone. It is especially appropriate for image-based assays because it measures the variability of intermediate responses to the assay, thus avoiding the possibility that the image analysis algorithms have been tuned to produce saturated results for just positive and negative controls.^29^ For both Z′ and V factors, the highest possible value (best assay quality) is 1, and they can range into negative values (for assays in which distinguishing between positive and negative controls is difficult or impossible). A Z′ factor >0 is potentially screenable; a Z′ factor >0.5 is considered an excellent assay.

We began by testing this approach on the cytoplasm-to-nucleus translocation (CNT) assay using the 2 publicly available image sets, BioImage and Vitra (**Fig. 1A**, top). In these assays, the relative distribution of fluorescence intensities between the cytoplasm and the nucleus of a cell changes under certain conditions. We measured this change by first correcting for illumination variations consistent across the image set in each channel (**Fig. 1B**). Using the DNA stain, we readily segmented the nuclei from the background. There is no separate stain to identify the cell boundary, so we identified 2 compartments of potential utility for the assay: (1) the region defined by the boundaries of the green fluorescence signal and (2) a compartment defined by dilating each nucleus a defined amount. We then took numerous measurements, including intensities, sizes, shapes, correlations between channels, radial distributions, and textures within each compartment, for each cell or across the entire image. For some features, we calculated ratios for each cell (e.g., intensity in the nucleus compartment vs. the dilated nucleus compartment), and for some features, we classified cells into categories above or below a few threshold values chosen by examining the values of features for particular samples using the *Show Data on Image* tool.

**Fig. 2.**
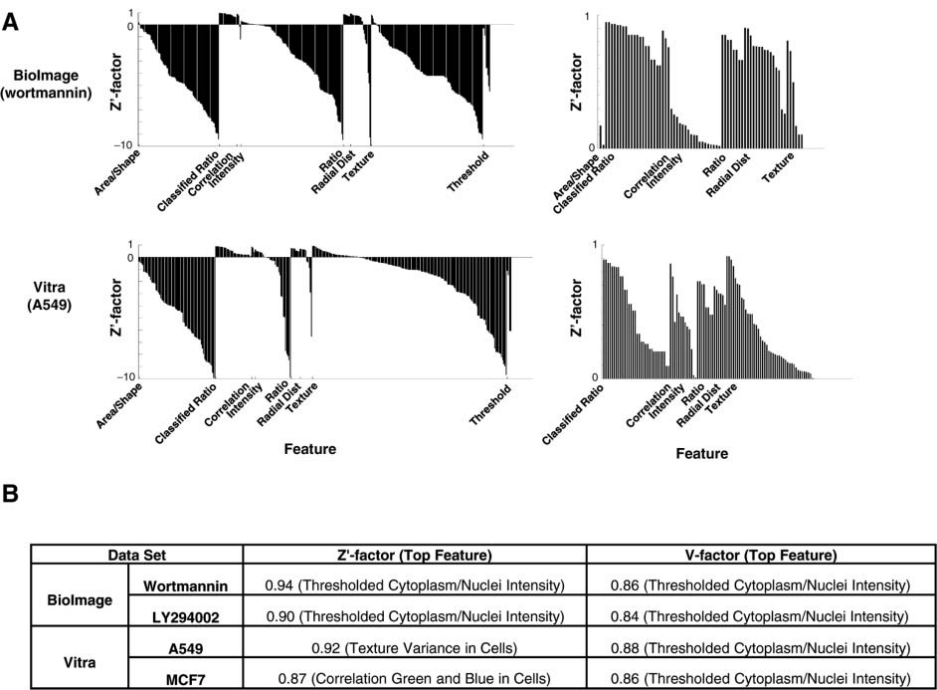
Cytoplasm-to-nucleus translocation (CNT) assay quality statistics. (**A, left**) Bar plots display Z′ factors for each feature, grouped by feature category and ordered by descending Z′ factor values within each category. Values below –10 are not displayed, and note that the maximum possible Z′ factor is 1. Feature categories begin at their *x*-axis label and progress to the right. BioImage LY294002 and Vitra MCF7 plots are not shown, as they are qualitatively similar to the respective plots shown. (**A, right**) The same data are plotted, but values below 0 are not shown, highlighting features that are potentially screenable. The 2 CNT data sets display similar screenable features, and V factors also display qualitatively the same pattern (not shown). Note that the number of intensity measures are greater in BioImage than in Vitra because the propagate algorithm was not reliable at detecting the cytoplasm in Vitra positive control images and thus not screened. (**B**) The top-scoring feature, in terms of Z′ and V factor, is shown for each CNT image set. BioImage has 2 drugs, wortmannin and LY294002, and Vitra has 2 cell types, A549 and MCF7.

For each measurement, the pipeline calculated statistical assay quality metrics, the Z′ and V factors, and we categorized these according to measurement category (**Fig. 2**, top). For the CNT assays, the highest Z′ and V factor categories (**Fig. 2B**) include the ratio between the mean intensities of the cytoplasm and nucleus compartments (*Ratio*), especially when thresholded at the per cell level (*Classified Ratio*), and all indicate that they are screenable assays (>0.5). These measurements were expected to be valuable and were in fact designed to suit the assay; thus, in this case, the value of the screening approach was in the ability to test several possible thresholds and dilation factors in the pipeline in parallel, speeding the identification of the most appropriate one for each particular image set. Still, it is worth noting that other measures such as *Correlation* (correlation of green and blue pixels), *Radial Distribution* (green pixel intensity distribution along a radius from cell centroid to dilated nucleus), and *Texture* (a spatial variance measure) are also effective readouts for this assay (**Fig. 2**, top right). Choosing one of these alternative measurements might be preferable in some cases. For example, several of these are less parameter dependent than the *Classified Ratio* feature (which required “tweaking” by setting a proper threshold that changes from experiment to experiment).

**Fig. 3.**
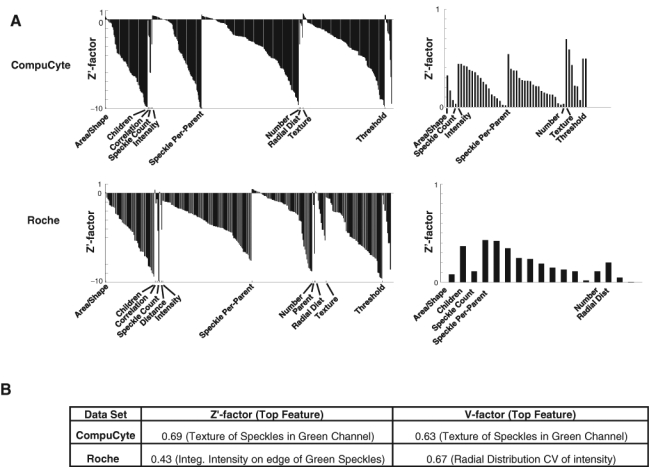
Transfluor assay quality statistics. Layout is the same as **Figure 2**. Note that values below –10 are not displayed, and this varied between the 2 data sets, explaining the seeming difference in numbers of features within each category.

We next analyzed the Transfluor assay, in which positive controls result in a speckled phenotype (**Fig. 1A**, bottom). We used pipelines similar to those for the CNT assay to extract measurements for the 2 Transfluor image sets, CompuCyte and Roche. We added some algorithms tailored for speckle identification to the pipeline, including the use of a top-hat filter in the *Enhance or Suppress Features* module and per object thresholding within *Identify Primary Objects*. The best categories for the Transfluor assays were mostly similar between them, although different from the CNT features. High-scoring features included *Speckle Count* (number of small, bright green spots per image), *Speckle Per Parent* (number of speckles per parent cell), *Texture* (CompuCyte), and *Radial Distribution* (Roche) (**Fig. 3A**). The top scoring V factors for each Transfluor set indicate that they are highly screenable (>0.5), although the Roche Z′ factor is slightly below this threshold (**Fig. 3B**). The top-scoring features encompassed a broad range, including speckle texture, integrated edge intensity of green speckles, and the coefficient of variation of intensity along a radial axis from center to cell membrane.

These results indicate that the screening approach is helpful for identifying those features that may be suited to score a particular assay in cases where machine learning is not preferred. There are a few cautionary notes about the approach to mention. First, selecting the best measure from a large library of measures can uncover spurious differences between positive and negative controls, particularly when only a small number of positive and negative controls are available. Therefore, the statistically sound approach is to select a feature based on one set of control samples (the “training set”) and then test whether that feature is also effective for scoring a separate set of control samples (the “test set”). Taking this approach with control samples prepared in different experimental batches is also good practice, to confirm both the statistical value of the selected image-based feature while simultaneously confirming biological reproducibility. The second danger of the screening approach for assay development feature selection is a hazard shared in common with machine-learning methods, as described in the Introduction. Features may be very effective for distinguishing a particular positive control from negative controls based on morphological properties that are not the primary goal of the assay. Take, for example, a cytoplasm-to-nucleus assay where the positive control induces translocation but also happens to reduce the growth rate of the cells. Cells would be less crowded in positive control images as compared to negative control images; care must be taken to avoid selecting the readout of the assay to be a feature that is simply a measure of cell sparseness rather than the true phenotype of interest. Some such features could be spuriously reading out phenotypes that are not of direct interest; for example, in some cell types, the intensity of a cytoskeletal stain at the edge of the cell will be higher if the cell touches another cell, yielding a perhaps surprising indirect measure of cell sparseness. Human intuition and an understanding of image analysis algorithms are often necessary to interpret whether a particular feature is measuring the true phenotype of interest. It is also helpful to use several positive control conditions that share the primary phenotype of interest but vary in other features of the cells, if such controls are available.

If processing speed is a concern, the methods described here can be used for assay development, and then the pipeline can be “dumbed down” to eliminate calculation of unnecessary measurements when running the assay in HTS mode. For some environments, streamlining the analysis may be helpful, but in practice, we rarely do this because we typically conduct extensive secondary analyses of the primary screening data to categorize hit samples by features other than the primary readout, thus harvesting as much information content from the primary screen as possible.

Here, we have demonstrated the usefulness of the screening approach to image-based assay development, whereby the best cellular feature for an assay is chosen quantitatively from a library of hundreds of candidate measurements. The concept is simple, and the methods built into CellProfiler make such an analysis straightforward, even for researchers without programming skills. All of the algorithms and software presented are open source and freely available. We presented results from the 4 publicly available image sets, the best published assay quality results to our knowledge. We are eager for other researchers to continue to advance the algorithms in this field by besting our results, publishing them, and ideally making open-source algorithms available for the screening community.
